# Unique Microbial Diversity and Metabolic Pathway Features of Fermented Vegetables From Hainan, China

**DOI:** 10.3389/fmicb.2018.00399

**Published:** 2018-03-06

**Authors:** Qiannan Peng, Shuaiming Jiang, Jieling Chen, Chenchen Ma, Dongxue Huo, Yuyu Shao, Jiachao Zhang

**Affiliations:** ^1^College of Food Science and Technology, Hainan University, Haikou, China; ^2^College of Food Engineering and Nutritional Science, Shaanxi Normal University, Xi' an, China; ^3^College of Food Science, Fujian Agriculture and Forestry University, Fuzhou, China

**Keywords:** fermented vegetables, high-throughput sequencing, microbial diversity, *Lactobacillus*, metabolic pathways

## Abstract

Fermented vegetables are typically traditional foods made of fresh vegetables and their juices, which are fermented by beneficial microorganisms. Herein, we applied high-throughput sequencing and culture-dependent technology to describe the diversities of microbiota and identify core microbiota in fermented vegetables from different areas of Hainan Province, and abundant metabolic pathways in the fermented vegetables were simultaneously predicted. At the genus level, *Lactobacillus* bacteria were the most abundant. *Lactobacillus plantarum* was the most abundant species, followed by *Lactobacillus fermentum, Lactobacillus pentosaceus*, and *Weissella cibaria*. These species were present in each sample with average absolute content values greater than 1% and were thus defined as core microbiota. Analysis results based on the alpha and beta diversities of the microbial communities showed that the microbial profiles of the fermented vegetables differed significantly based on the regions and raw materials used, and the species of the vegetables had a greater effect on the microbial community structure than the region from where they were harvested. Regarding microbial functional metabolism, we observed an enrichment of metabolic pathways, including membrane transport, replication and repair and translation, which implied that the microbial metabolism in the fermented vegetables tended to be vigorous. In addition, *Lactobacillus plantarum* and *Lactobacillus fermentum* were calculated to be major metabolic pathway contributors. Finally, we constructed a network to better explain correlations among the core microbiota and metabolic pathways. This study facilitates an understanding of the differences in microbial profiles and fermentation pathways involved in the production of fermented vegetables, establishes a basis for optimally selecting microorganisms to manufacture high-quality fermented vegetable products, and lays the foundation for better utilizing tropical microbial resources.

## Introduction

Vegetables are a primary source of nutrition for the human body, as they provide a variety of necessary vitamins and minerals. In addition to being cooked, vegetables can be pickled, dried, canned, frozen, and converted into liquid forms. These processes extend their storage life, facilitate their transport, increase their value, and improve their flavor. In addition, they can also increase the variety of vegetable products to meet the growing demand for vegetable foods.

Vegetable fermentation is a way of processing vegetables, using vegetables as raw materials, and harnessing beneficial microorganisms, but the process requires certain production conditions. The vegetable fermentation system comprises a microecological environment that contains a variety of microorganisms, such as lactic acid bacteria, yeast, and acetic acid bacteria. Of all the microorganisms involved in vegetable fermentation, lactic acid bacteria are the predominant genera utilized for the fermentation process (Nguyen et al., 2013). The flavor of a vegetable after fermentation is closely related to the flavor of the fresh vegetable itself, its microbial metabolic activity and its fermentation conditions (Petaja et al., 2000; Liu and Tong, 2017). Approximately 3,000 years ago, the Chinese discovered the vegetable fermentation process, using it to produce Sichuan pickles and hot pickled mustard tubers, among others. With the recent rapid development of the fermented food industry, fermented products have enriched the diversity of the human diet with their excellent flavors and tastes. Lactic acid bacteria can attach to the surface of vegetables, fermenting sugar, and other nutrients to produce lactic acid. Furthermore, fermented vegetables have the nutritional and healthy functions necessary to adjust the balance between intestinal microbiota and lower cholesterol, making these vegetables essential for consumers (Gallartjornet et al., 2007). Studies on microorganisms found in fermented vegetables have been previously reported. In the 1960s, Albury (1953) and Pederson and Albury (1961) studied the fermentation of sour cucumber and cabbage, finding that *Leuconostoc mesenteroides, Pediococcus cerevisiae, Lactobacillus brevis* and *Lactobacillus plantarum* were active in the fermentation process. Several strains with excellent performance have also been isolated and screened from Chinese pickles, including *Lactobacillus plantarum, Leuconostoc mesenteroides, Leuconostoc mesenteroides, Lactobacillus casei, Lactobacillus brevis* and *Lactobacillus acidophilus*. Jiang et al. (2012) observed that *Lactobacillus sakei, Lactobacillus plantarum, Lactobacillus brevis* and *Leuconostoc mesenteroides* are single- or multiple-dominant lactic acid bacteria in fermented radish of China. *Lactobacillus crustorum, Lactobacillus farciminis*, and *Lactobacillus mindensis* were isolated as important microorganisms in Taiwanese mustard products by Chao et al. (2012). (Yu et al., 2012) isolated 185 lactic acid bacteria from pickles collected from six regions of Sichuan Province and accurately identified 81 *Lactobacillus plantarum*, 38 *Lactobacillus pentosus*, and 24 *Lactobacillus brevis*. Cho et al. (2006) studied the fermentation process of kimchi and demonstrated that kimchi fermentation is governed by the distinct population dynamics of a subset of *Lactobacillus, Leuconostoc*, and *Weissella* species, such as *Leuconostoc citreum* and *Weissella cibaria*. These studies demonstrated that the microbial diversities of traditional fermented foods are abundant.

Hainan Province is an island located at the southern tip of China that has a tropical monsoon climate with a long summer and no winter, abundant sunshine and good rainfall. Historically, Hainan is a gathering place for ethnic minorities. This province supports tropical agricultural, and its animal and plant resources are abundant. Due to its specific microbial resources (because of its island geography), Hainan has become an important region for the development of tropical biotechnology and tropical resource studies. The populates of Hainan have inhabited the island for generations and have used rich microbial resources to produce various fermented foods for a long time. Fermented vegetables are generally welcomed by a vast number of consumers as a kind of traditionally fermented food with high nutritional value, and have naturally become the best model for studying the microbial diversities of tropically fermented foods.

In our previous research, we studied the microbial diversity of Yucha, a traditionally fermented cereal of Hainan Province (Zhang et al., 2016). We found that the diversity of microbial resources in Yucha was high, and the microbial metabolism was vigorous. To reveal the abundant microbial diversities and microbial community structures and explore the metabolic pathways in fermented vegetables of Hainan, we investigated fermented vegetable samples collected from different regions of Hainan Province using high-throughput sequencing and culture-dependent technology. The present study should facilitate our understanding of differences in the microbial profiles of fermrnted vegetables and the fermentation pathways involved in their production, thereby helping to establish a basis for optimally selecting microorganisms for the manufacture of high-quality fermented vegetable products and laying the foundation for better utilizing tropical microbial resources.

## Results

### Comparative analysis and sequencing of the fermented vegetable samples

Information on fermented vegetable samples and sequencing results are shown in Table 1, and the specific region and type of samples are shown in Supplementary Table S1. We analyzed each indicator of fermented vegetable samples from both the regional and species perspective. Using high-throughput sequencing, we generated a dataset containing 267,422.22 filtered, high-quality and classifiable 16S rRNA gene sequences, with an average of 66,873.23 sequences per sample (range: 64,346.89–69,396.64). All sequences were clustered with a 97% sequence identity cut-off, and a total of 1,492.70 OTUs were filtered for further analysis, with an average of 373.175 OTUs per sample (range: 225.33–581.80). The average read lengths were between 425.9 and 427.67 bases, and Q20 values ranged from 98.35 to 98.37, indicating that the quantities and qualities of the sequences were sufficient. At high altitudes in the midline area, the average pH of the samples was 3.73, while the highest average pH was in the east line samples. Fermented Chinese cabbages were located at the lowest altitude among the different types of fermented vegetables and had the highest average pH and lowest TTA and lactic acid contents.

**Table 1:** Sample information and sequencing results.

**Group**	**Samples**	**pH**	**TTA (ml)**	**Lactic acid**	**Qualified**
Region	ES	3.92	13.58	15.96	68939.80
	MS	3.73	15.67	19.82	65386.27
	WS	3.57	16.60	22.75	66364.33
Species	FBS	3.56	16.64	24.08	64346.89
	FCC	3.89	13.81	16.62	69396.64
	FGB	3.77	15.45	19.15	65365.36
	FW	3.54	17.03	22.15	68313.33
**Group**	**Avg-Len**	**OTU#**	**Elevation**	**Temp**	**Humidity**
Region	425.90	581.80	15.50	23.95	0.82
	427.45	337.82	295.55	22.45	0.83
	427.33	292.13	182.00	23.50	0.78
Species	427.67	225.33	176.39	23.11	0.79
	426.00	566.09	97.32	23.83	0.80
	427.18	355.45	216.18	22.86	0.83
	427.50	345.83	238.08	23.28	0.79

### Alpha diversity in the fermented vegetable samples

**Figure 1:**
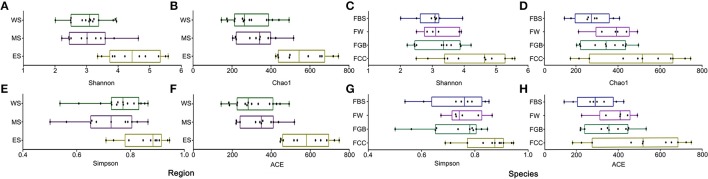
Differences in microbial alpha diversities among the fermented vegetables samples. **(A,B,E,F)** Microbial alpha diversity among samples in different regions. **(C,D,G,H)** Microbial alpha diversity among samples of different vegetable species.

The alpha diversities of microbes from different regions and different vegetable species, measured via the Shannon, Chao1, Simpson and ACE indices, were compared using the QIIME platform (Figure 1). Among the fermented vegetables collected from different regions, the alpha diversity of the samples collected from the east line was the best, yielding the highest Shannon, Chao1, Simpson and ACE index values. The various index values of fermented cabbage were significantly higher than those of the other three fermented vegetables, implying that the microbial diversity and uniformity of fermented cabbage was the highest. Supplementary Figures S1, S2 show that the alpha diversity of fermented cabbage in different types of fermented vegetables in the same area was the best, and the alpha diversity of samples collected from the east line in the same type of fermented vegetables in different regions was higher than that of the others. These results showed that significant differences in alpha diversity existed among the fermented vegetable samples collected from different regions and different vegetable species.

### Microbial diversity in the fermented vegetable samples

**Figure 2:**
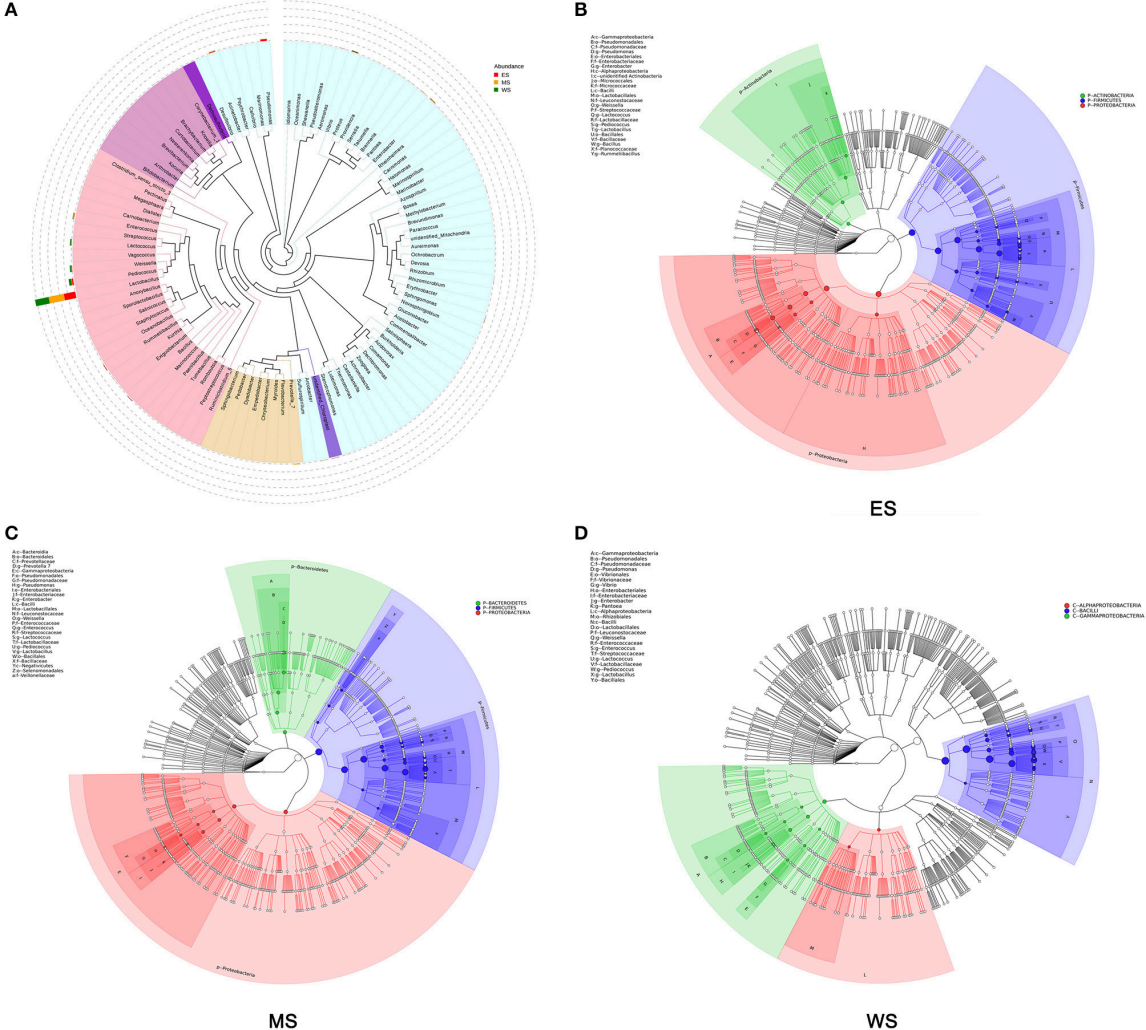
Landscape of microbial diversity in the fermented vegetables. **(A)** Gross sample. **(B)** East line sample. **(C)** Midline sample. **(D)** West line sample.

The microbial diversities of the fermented vegetables were described from the perspectives of each region (ES, MS, and WS) and all regions in combination. The evolutionary relationship of each microbe is shown in Figure 2A. *Lactobacillus, Pediococcus* and *Weissella* comprised the top three genera, and *Lactobacillus* was the most abundant genus. Regionally, *P-Firmicutes, P-Proteacteria*, and *P-Actinobacteria* were the most abundant in the east line samples (Figure 2B); *P-Firmicutes, P-Bacteroidetes*, and *P-Proteobacteria* were the most abundant in the midline samples (Figure 2C); and *C-Bacilli, C-Gammaproteobacteria*, and *C-Alphaproteobacteria* were the most abundant in the west line samples (Figure 2D). *Lactobacillus* was consistently the most abundant genus in the three regions.

### Microbial composition and core microbiota of the fermented vegetables samples

**Figure 3:**
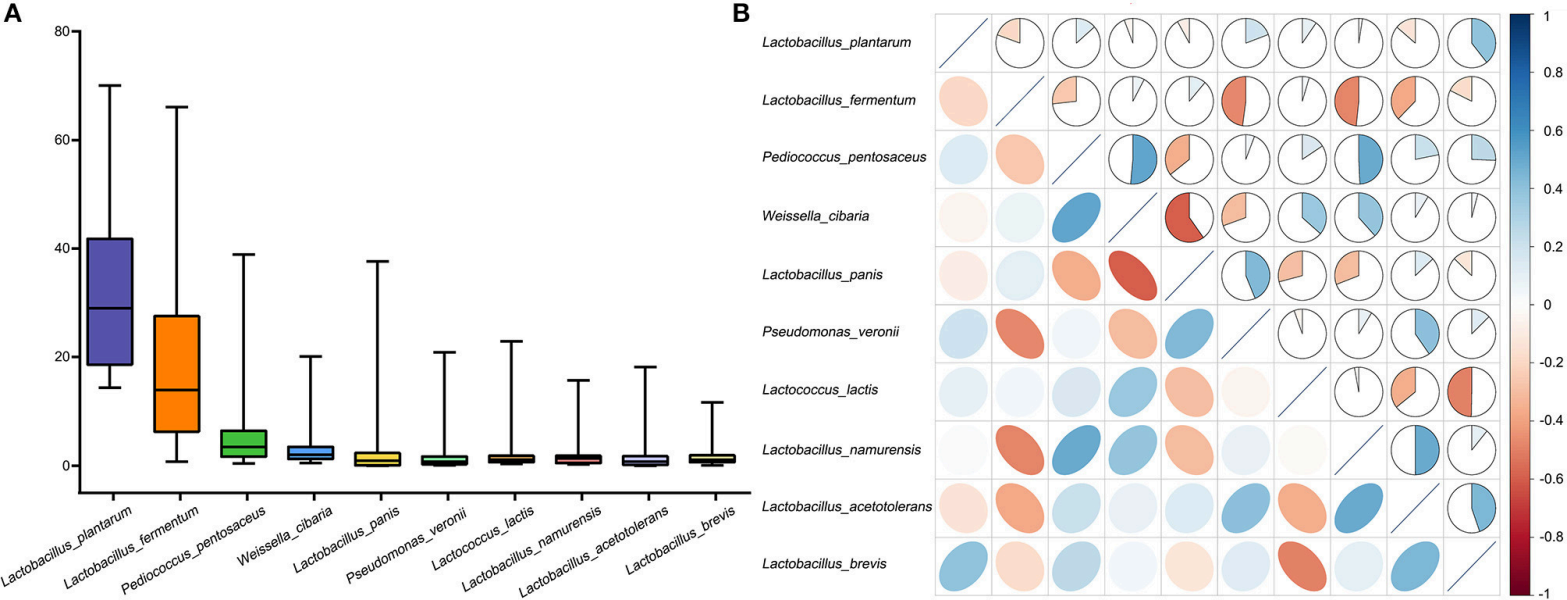
Compositions of microbiota in the fermented vegetables samples. **(A)** Box-plots showing the amounts of predominant bacteria as quantified by q-PCR. **(B)** Correlation matrix showing the Spearman's rank correlation among the 10 core species. The Spearman's rank correlation coefficients ranged from 1.0 to −1.0, corresponding to strongly positive correlations to strongly negative correlations.

We quantified the predominant microbiota in the fermented vegetables samples and found that the quantities of *Lactobacillus plantarum, Lactobacillus fermentum, Lactobacillus pentosaceus, Weissella cibaria, Lactobacillus panis, Pseudomonas veronil, Lactococcus lactis, Lactobacillus namurensis, Lactobacillus acetotolerans*, and *Lactobacillus brevis* all exceeded 1% (Figure 3A). We thus defined these species as core microbiota. Among them, *Lactobacillus plantarum* was the most abundant at 32.31%. Correlations among the core microbiota were also determined using Spearman's rank correlation analysis (Figure 3B). A negative correlation was found between *Lactobacillus plantarum* and *Lactobacillus brevis*, while a positive correlation was found between *Lactobacillus plantarum* and *Lactobacillus panis*.

### Beta diversity in the fermented vegetable samples

**Figure 4:**
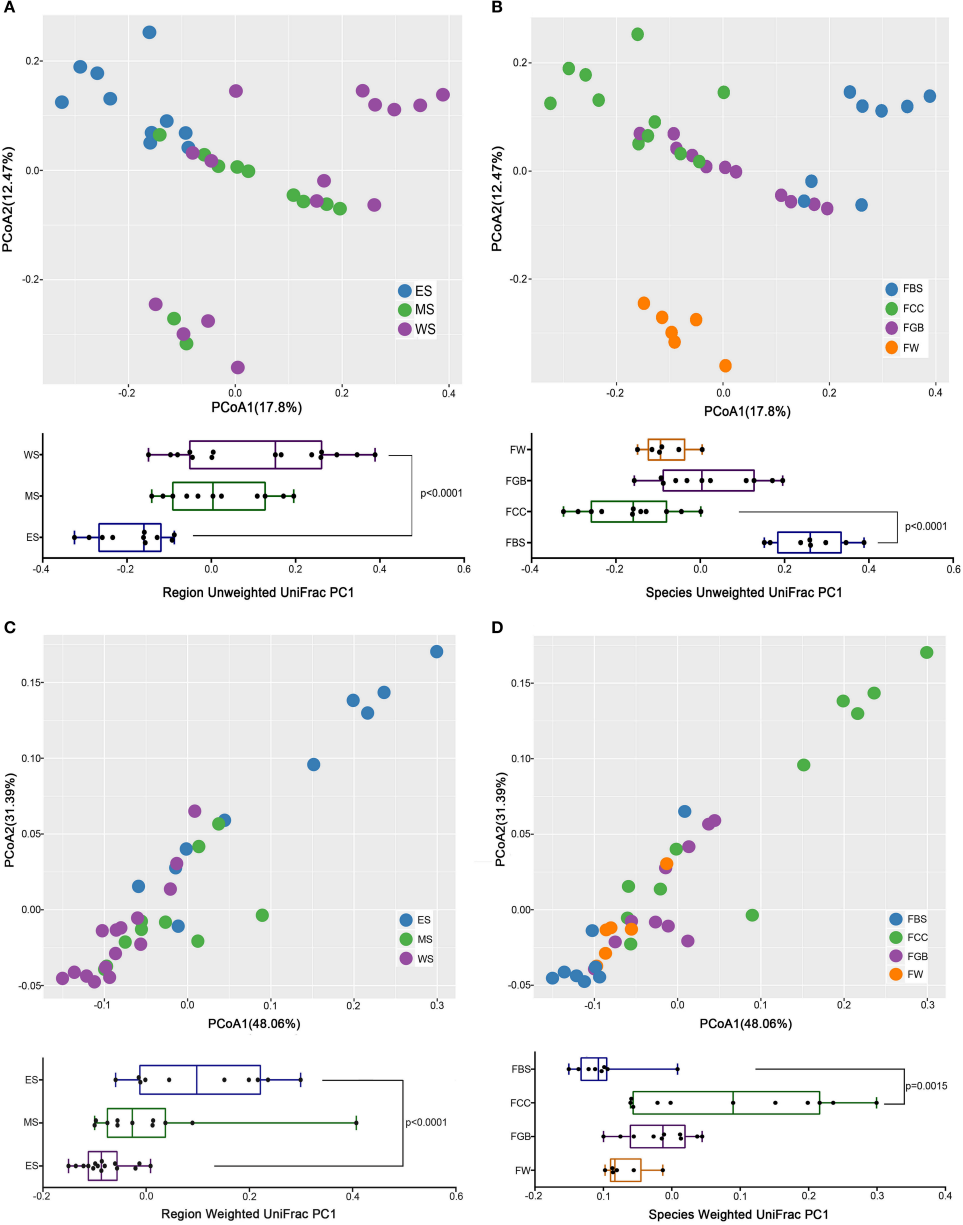
Microbial beta diversity among the fermented vegetables samples. **(A)** Region Unweighted Unifrac. **(B)** Species Unweighted Unifrac. **(C)** Region Weighted Unifrac. **(D)** Species Weighted Unifrac.

To analyse the beta diversities of the fermented vegetable samples, we compared the Weight UniFrac and Unweight Unifrac distances among fermented vegetable samples collected from different regions and different vegetable species. As shown in Figure 4, the microbial community structures in different regions were highly mixed and difficult to distinguish, while the microbial structural difference among the types of fermented vegetables was significant and distinct. Supplementary Figures S3, S4 show that microbial community structures within the same type of fermented vegetables in different regions have a higher degree of confoundment than those within different types of fermented vegetables in the same region. Thus, we concluded that the vegetable species had a greater impact on the microbial community than the region wherein the sample was collected.

**Figure 5:**
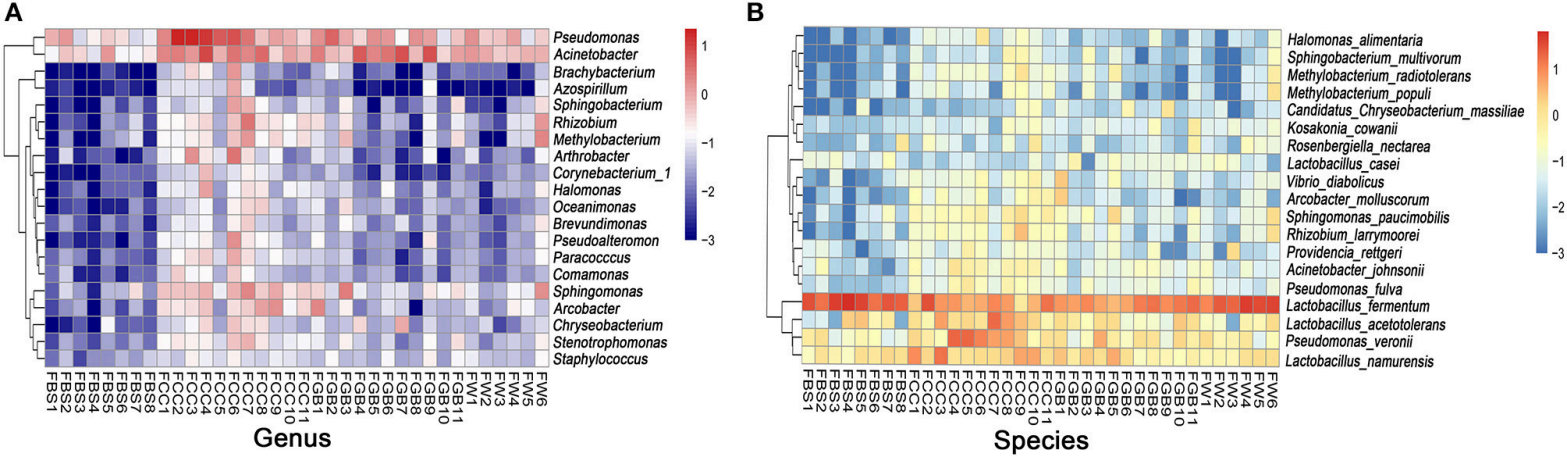
Microbial differences at the genus/species level. **(A)** Genus level. **(B)** Species level.

Next, we identified the differences in bacteria at the genus and species levels in different species (Figure 5) and regions (Supplementary Figures S5, S6). As shown in Figure 5, the quantity of *Chryseobacterium* in fermented bamboo shoots was significantly lower than that in the other groups, whereas the contents of *Sphingomonas* and *Pseudomonas* in fermented Chinese cabbage were significantly higher than those in other groups. Additionally, *Lactobacillus fermentum* was enriched in each group. The quantities of *Sphingomonas paucimobiliz* and *Rhizobium larrymoorei* in fermented Chinese cabbage were higher than those in the other groups, whereas the quantities of *Acinetobacter johnsonii* and *Pseudomonas fulva* in fermented bamboo shoots were lower than those in the other groups.

### Identification of *Lactobacillus* isolates

Using culture-dependent technology, 353 *Lactobacillus* strains were isolated from 36 fermented vegetable samples. The molecular identification results based on 16S rRNA gene sequencing are shown in Table 2. *Lactobacillus plantarum* (123 isolates) and *Lactobacillus fermentum* (116 isolates) existed in nearly all of the fermented vegetable samples from each region and were the predominant *Lactobacillus* species in fermented vegetables from Hainan. Additionally, *Enterococcus faecalis* and *Lactobacillus nantensis* were isolated from only samples collected from Wanning, *Lactobacillus nasuensis* was isolated from only the Wuzhishan group, and *Lactobacillus suntoryeus* was isolated from only the Changjiang group.

**Table 2:** Identification of *Lactobacillus* isolates based on 16S rDNA sequencing.

**Identification**	**Samples**						
	**WC**	**WN**	**QZ**	**WZS**	**BS**	**CJ**	**LG**
*Enterococcus faecalis*	–	2/2	–	–	–	–	–
*Enterococcus faecium*	–	4/48	18/48	13/48	13/48	–	–
*Lactobacillus acetotolerans*	–	3/3	–	–	–	–	–
*Lactobacillus alimentarius*	–	3/3	–	–	–	–	–
*Lactobacillus brevis*	–	–	1/9	2/9	1/9	3/9	2/9
*Lactobacillus composti*	–	–	–	2/2	–	–	–
*Lactobacillus coryniformis*	–	–	–	1/1	–	–	–
*Lactobacillus delbrueckii*	–	1/1	–	–	–	–	–
*Lactobacillus fermentum*	–	6/116	20/116	19/116	13/116	33/116	25/116
*Lactobacillus futsaii*	–	1/1	–	–	–	–	–
*Lactobacillus nagelii*	1/2	–	–	–	–	–	1/2
*Lactobacillus namurensis*	–	1/14	–	6/14	–	2/14	5/14
*Lactobacillus nantensis*	–	3/3	–	–	–	–	–
*Lactobacillus nasuensis*	–	–	–	1/1	–	–	–
*Lactobacillus oris*	–	–	–	–	1/1	–	–
*Lactobacillus panis*	–	1/3	–	–	1/3	1/3	–
*Lactobacillus parafarraginis*	–	1/1	–	–	–	–	–
*Lactobacillus pentosus*	–	–	–	–	1/4	1/4	2/4
*Lactobacillus plantarum*	3/123	8/123	40/123	18/123	12/123	38/123	4/123
*Lactobacillus senioris*	–	1/1	–	–	–	–	–
*Lactobacillus senmaizukei*	–	–	–	1/1	–	–	–
*Lactobacillus shenzhenensis*	–	–	–	2/2	–	–	–
*Lactobacillus silagei*	–	–	–	2/2	–	–	–
*Lactobacillus suntoryeus*	–	–	–	–	–	1/1	–
*Lactobacillus vini*	2/6	2/6	–	–	2/6	–	–
*Lactobacillus zymae*	1/2	1/2	–	–	–	–	–

### Comparison of metabolic pathway abundances

**Figure 6:**
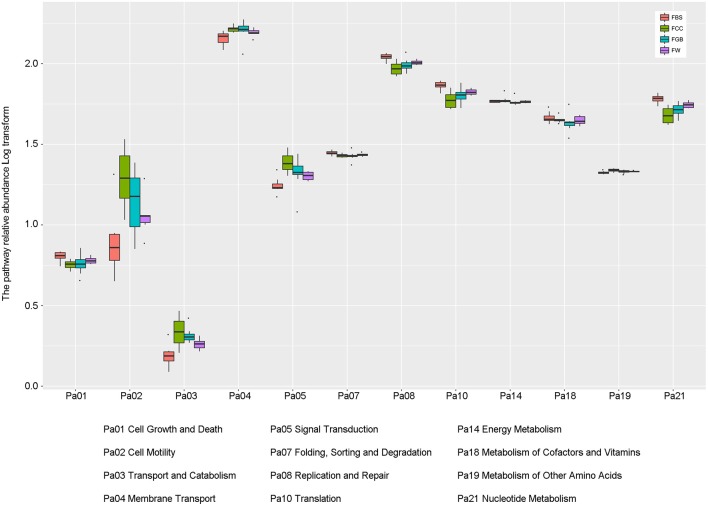
Abundances of metabolic pathways in different types of fermented vegetables.

To better understand the important roles of specific microbes in the fermented vegetables, the levels of various metabolic pathways among the different types of fermented vegetable samples were compared. As shown in Figure 6, the metabolic pathways in the fermented vegetable samples of Hainan were abundant, implying that microbial metabolism in the fermented vegetables tended to be vigorous. Among these metabolic pathways, three metabolic pathways, membrane transport, replication and repair and translation, were the most abundant in all species of the fermented vegetable samples. Meanwhile, the contribution rates of the core microbiota to the various metabolic pathways related to microbial metabolism were calculated (Table 3). *Lactobacillus plantarum* and *Lactobacillus fermentum* were the main metabolic pathway contributors, contributing 10.417 and 5.837% to amino acid metabolism and 13.106 and 7.344% to carbohydrate metabolism, respectively.

**Table 3:** Contribution rates (%) of the predominant species to metabolism pathways.

**Species**	**A**	**B**	**C**	**D**	**E**	**F**	**G**	**H**	**I**	**J**	**K**	**H**
*Lactobacillus plantarum*	10.417	0.758	13.106	5.847	2.423	2.378	4.024	4.455	2.148	2.295	5.324	3.310
*Lactobacillus fermentum*	5.837	0.425	7.344	3.276	1.358	1.333	2.255	2.496	1.203	1.286	2.983	1.855
*Pediococcus pentosaceus*	1.775	0.129	2.233	0.996	0.413	0.405	0.686	0.759	0.366	0.391	0.907	0.564
*Weissella cibaria*	0.992	0.072	1.248	0.557	0.231	0.226	0.383	0.424	0.205	0.219	0.507	0.315
*Lactobacillus panis*	0.844	0.061	1.062	0.474	0.196	0.193	0.326	0.361	0.174	0.186	0.432	0.268
*Pseudomonas veronii*	0.817	0.059	1.028	0.458	0.190	0.186	0.315	0.349	0.168	0.180	0.417	0.260
*Lactococcus lactis*	0.725	0.053	0.913	0.407	0.169	0.166	0.280	0.310	0.150	0.160	0.371	0.230
*Lactobacillus namurensis*	0.686	0.050	0.863	0.385	0.160	0.157	0.265	0.294	0.142	0.151	0.351	0.218
*Lactobacillus selangorensis*	0.633	0.046	0.796	0.355	0.147	0.144	0.244	0.271	0.130	0.139	0.323	0.201
*Lactobacillus acetotolerans*	0.552	0.040	0.694	0.310	0.128	0.126	0.213	0.236	0.114	0.122	0.282	0.175
*Lactobacillus brevis*	0.518	0.038	0.651	0.291	0.120	0.118	0.200	0.221	0.107	0.114	0.265	0.165

*A, Amino Acid Metabolism; B, Biosynthesis of Other Secondary Metabolites; C, Carbohydrate Metabolism; D, Energy Metabolism; E, Enzyme Families; F, Glycan Biosynthesis and Metabolism; G, Lipid Metabolism; H, Metabolism of Cofactors and Vitamins; I, Metabolism of Other Amino Acids; J, Metabolism of Terpenoids and Polyketides; K, Nucleotide Metabolism; H, Xenobiotics Biodegradation and Metabolism*.

### Correlation analysis of core microbiota and metabolic pathways

**Figure 7:**
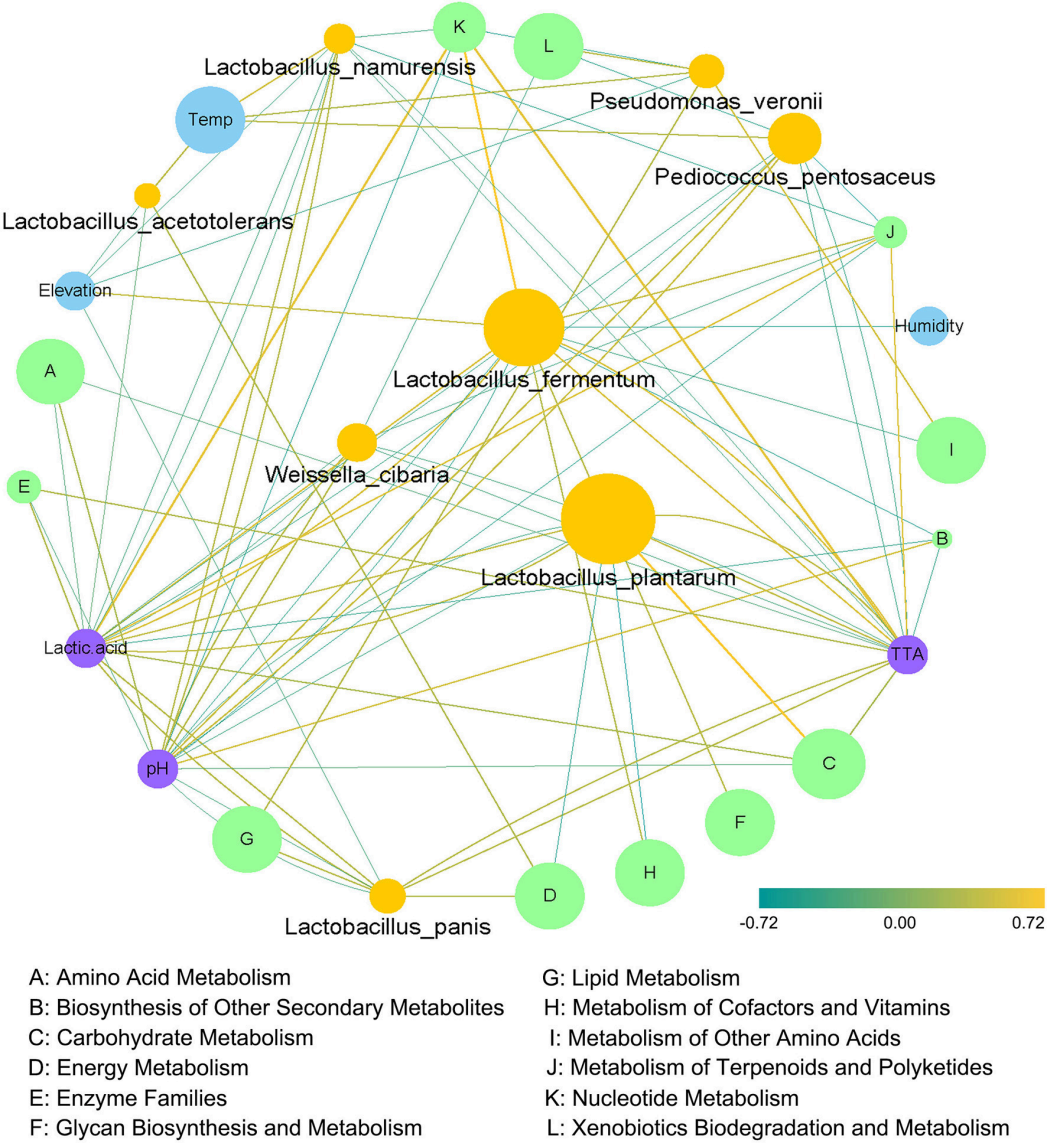
Correlation network constructed based on climatic conditions, bacterial species, microbial metabolic pathways and physicochemical indices.

We further explored correlations among the core microbiota, metabolic pathways, geographical conditions and physicochemical indices using Spearman's rank correlation coefficient analysis. As shown in Figure 7, a generally positive correlation was observed between *Lactobacillus plantarum*, the most abundant species in the fermented vegetables, and carbohydrate metabolism, and a negative correlation was found between *Lactobacillus plantarum* and the metabolism of cofactors and vitamins. Meanwhile, a general positive correlation was observed among *Lactobacillus fermentum*, nucleotide metabolism and lactic acid. Additionally, a significant positive correlation was found between nucleotide metabolism and TTA, and a significant negative correlation was found between nucleotide metabolism and pH.

## Discussion

In this study, high-throughput sequencing and culture-dependent technology were used in combination to study the microbial communities and metabolic pathways of fermented vegetables in Hainan. We observed that *Lactobacillus* was the most abundant genus, and *Lactobacillus* metabolism was the most vigorous. *Lactobacillus* are Gram-positive bacteria that ferment glucose and lactose to lactic acid and are beneficial to human health. The abundant *Lactobacillus* found in our samples likely originated on the vegetable surfaces and surrounding environment and played multiple roles during fermentation. After harvesting, vegetable surfaces generally contain many microorganisms, although the number of beneficial microorganism is generally low. With the addition of salt to fermented vegetable processing, salt-sensitive microorganisms fail to grow, and reproduce. *Lactobacillus* has a high salt tolerance (Yang and Ling, 1999) and can ferment sugars in vegetables to produce acid, creating an acidic environment to inhibit the growth of other bacteria. In addition, a variety of antimicrobial substances produced by lactic acid bacteria via the metabolization of raw material components (Nguyen et al., 2013),such as hydrogen peroxide, diacetyl, Nisin, carbon dioxide, and bacteriocin, can inhibit the growth of spoilage and pathogenic bacteria (Kang et al., 2002; Cizeikiene et al., 2013). The vegetables themselves also contain certain chemicals that selectively inhibit microorganisms other than *Lactobacillus*, such as isothiocyanates and allyl disulphide. Additionally, the vegetable fermentation system is an anaerobic environment, and some aerobic microorganisms, including spoilage bacteria, are difficult to grow in fermented vegetables (Yan and Xue, 2005). Therefore, the high-salt, high-acid, antimicrobial, and anaerobic environment primarily selects for the beneficial anaerobic *Lactobacillus*.

During fermentation, microorganisms convert vegetables into products of increased value. Although *Lactobacillus* comprise only a small fraction of the plant autochthonous microbiotic community, they dominate the vegetable fermentation process and represent important microbes that can impact the health-promoting properties of plant foods. Plants contain various anti-nutritional factors and are excellent sources of health-promoting components. Due to the inherent chemical component of the plants themselves and the metabolic and functional diversities of *Lactobacillus, Lactobacillus* bacteria maintain a variety of metabolic pathways, which appear as a complex network (Filannino et al., 2017). Each network pathway involves unique bacterial enzymes for specific substrates. The synergistic effect of specific *Lactobacillus* metabolic features and plant enzyme activities may increase the bioavailability and bioactivity of phytochemicals, potentially resulting in a significantly increased number of functional microbial metabolites, which may have beneficial effects on human health (Filannino et al., 2017). The fermentation of plant foods requires *Lactobacillus*, which are vital to the bioconversion of natural plant ingredients via different functional and metabolic pathways. *Lactobacillus* are indispensable bacteria that have important physiological functions in humans and animals, and they can regulate the microecological balance in the intestinal tract (Li and Chen, 2007). In addition to helping absorb the nutrient contents of fermented vegetables, *Lactobacillus* and their metabolites can inhibit the growth of spoilage bacteria in the intestinal tract, promote the absorption of nutrients, alleviate lactose intolerance, improve intestinal tract function, prevent constipation, reduce serum cholesterol and blood lipid levels, and adjust human physiological functions. Additionally, *Lactobacillus* may improve the quality of fermented vegetables by increasing their nutritional value, improving their flavor, inhibiting microbial growth, and reducing nitrite accumulation as well as the accumulation of other harmful substances during vegetable fermentation (Cizeikiene et al., 2013).

In this study, we performed in-depth profiling and characterization of the microbiomes of fermented vegetables samples collected from different regions of Hainan Province and simultaneously predicted the metabolic pathways in the fermented vegetables. We confirmed that *Lactobacillus* bacteria were the most abundant species during tropical vegetable fermentation, and *Lactobacillus plantarum* was the most abundant species, followed by *Lactobacillus fermentum, Lactobacillus pentosaceus* and *Weissella cibaria*. These species were present in each sample with average absolute content values greater than 1% and were thus defined as core microbiota. The analysis of microbial communities demonstrated that the microbial profiles of the fermented vegetables differed significantly based on the region and raw materials used; the alpha diversity of samples collected from the east line of Hainan Province was the best, and the microbial diversity and uniformity of fermented cabbage was the highest. Furthermore, the vegetable species had a greater effect on the microbial communities than the region wherein they were collected, and the genera and species of microbial communities in the different types of fermented vegetable samples were also identified. Moreover, we predicted the metabolic pathways related to specific microbes in the fermented vegetables samples from Hainan, observing an enrichment in some metabolic pathways, including membrane transport, replication and repair and translation, which implied that the microbial metabolism in the fermented vegetables tended to be vigorous. Further, we calculated the contribution rates of the core microbiota in the various metabolic pathways, revealing that *Lactobacillus plantarum* and *Lactobacillus fermentum* are the major metabolic pathway contributors. Finally, because the composition of microbes in fermented vegetables is influenced by many factors, correlations among the core microbiota, metabolic pathways, geographical conditions and physicochemical indices were studied. A general positive correlation was found among *Lactobacillus fermentum*, nucleotide metabolism and lactic acid. Meanwhile, a significant negative correlation was observed between nucleotide metabolism and pH. Additionally, a significant positive correlation was found between *Lactobacillus plantarum*, the most abundant species in the fermented vegetables, and carbohydrate metabolism. The present study investigated the tropical microbial resources of fermented vegetables in Hainan. We aimed to facilitate our understanding of differences in the microbial profiles of fermented vegetables and how fermentation pathways are involved in their production, thereby helping to eatablish a basis for selecting microorganisms to optimally manufacture high-quality fermented vegetable products. Additionally, our study also lays the foundation for better utilizing tropical microbial resources.

## Material and methods

### Collection and chemical analysis of the fermented vegetable samples

Thiety-six fermented vegetable samples [including fermented Chinese cabbages (FCC), fermented green beans (FGB), fermented bamboo shoots (FBS), and fermented watermelons (FW)] were aseptically collected from different regions of the Hainan Province in China [including the east line area (Wanning, Wenchang), the west line area (Lingao, Changjiang, and Baisha), and the midline area (Qiongzhong and Wuzhishan)]. The samples were refrigerated at 4°C before being transferred to the laboratory. The pH and lactic acid contents were determined, and the total titratable acidity values were measured in a relatively short time according to previous methods (Cavalcanti et al., 2010).

### DNA extraction and PCR amplification

Ten milliliters of each fermented vegetable sample was mixed with 90 mL of sterile NaCl solution (0.85%, w/v) and then homogenized into a homogenous suspension for 10 min. The QIAGEN DNA Mini-Kit (QIAGEN, Hilden, Germany) and bead-beating method were used in combination to extract DNA from the suspension. The integrity and purity of the extracted DNA were evaluated by 0.8% agarose gel electrophoresis, and the OD 260/280 value was measured using a micro-ultraviolet spectrophotometer to determine the DNA concentration. All extracted DNA samples were stored at −20°C.

Amplification of theV3-V4 regions of the 16S ribosomal RNA (rRNA) genes of each sample was performed. A set of 6-nucleotide barcodes was added to the universal forward primer 338F (5′-ACTCCTACGGGAGGCAGCA-3′) and the reverse primer 806R (5′-GGACTACHVGGGTWTCTAAT-3′) (Tanaka et al., 2009; Dethlefsen and Relman, 2011).

### Quantification, pooling and sequencing of the PCR products

The Agilent DNA 1000 Kit and Agilent 2100 Bioanalyser (Agilent Technologies, USA) were used in combination to quantify the PCR products according to the manufacturer's instructions. The amplified products of all the samples were pooled to a final concentration of 100 nmol/L in equimolar ratios and then loaded onto the Illumina MiSeq high-throughput sequencing platform for sequencing.

### Isolation and identification of *Lactobacillus*

Ten milliliters of each fermented vegetable sample was mixed with 90 mL of sterile NaCl solution (0.85%, w/v), homogenized into a homogenous suspension, and then subjected to a 10-fold serial dilution. Next, 100 μl of each dilution sample was spread onto MRS (de Man, Rogosa and Sharp; Difco, Detroit, MI, USA) agar medium using a spreading plate and incubated at 37°C under anaerobic conditions for 2 days to isolate *Lactobacillus*. After observing and recording the colony morphology, suspicious colonies were picked from the medium of optimal dilution and inoculated into the corresponding broth medium at 37°C for 24 h. Cell morphologies were observed under a light microscope by Gram staining to determine whether the pure bacteria were pure.

DNA was extracted from isolated single strains according to the method provided by Andrighetto et al. (2001). PCR amplification was performed according to previous reports (Liu et al., 2012). The extracted DNA was used as a template, and the primers A27F (5′-AGCGGATCACTTCACACAGGACTACGGCTACCTTGTTACG-3′) and A1495R (5′-GCAGAGTTCTCGGAGTCACGAAGAGTTTGATCCTGGCTCA-3′) were used to amplify the 16S rRNA gene. The PCR products (approximately 1,500 bp) were sequenced by Majorbio Corporation (Shanghai) after amplification.

The sequencing results were aligned using SeqMan software, and the obtained sequences were then compared with those in the NCBI database to determine sequence homology. We constructed a phylogenetic tree with the MEGA programme using the adjacency distance method and bootstrap analyses based on 1,000 random re-samplings to determine the confidence values of each branch.

### Bioinformatics and statistical analyses

The original sequences were trimmed to remove the low-quality sequences. The sliding window method was used to filter the sequences according to quality scores. After filtering, more than 85.3% of the sequences were retained. The QIIME (Caporaso et al., 2010b) analysis platform was used to perform bioinformatics analyses on the extracted high-quality sequences before and after the removal of primers and labels. PyNAST (Caporaso et al., 2010a) was used to align the sequences, and Uclust (Edgar, 2010) was used to cluster them under 100% sequence identity to obtain the unique V3-V4 sequence set. After representative sequences were selected, the operational taxonomic unit (OTU) was established with a clustering of 97% similarity. After the removal of chimeric sequences present in the OTU sequences, homologous analysis and taxonomic identification of the residue sequences were performed using the Ribosomal Database Project (RDP) (Cole et al., 2007) classifier with a minimum bootstrap threshold of 80%. The remaining representative sequences were inserted into Fast Tree (Price et al., 2009) software to generate phylogenetic trees from the represented sequences. Alpha and beta diversity calculations were performed on this basis. The Shannon, Chao1, Simpson, and ACE indices were calculated to evaluate alpha diversity, and UniFrac (Lozupone and Knight, 2005) metrics were calculated to evaluate beta diversity. Weighted and unweighted principal coordinate analyses (PCoA), based on the UniFrac distance, were performed between samples, and the unweighted pair-group method with arithmetic means (UPGMA), based on UniFrac, was used for sample clustering. PICRUSt was used to predict functional features of the 16S rRNA based on the high-throughput sequencing data (Langille et al., 2013).

The R programme was used for statistical analysis. The relative abundances of taxa were compared using the Kruskal-Wallis test based on the rarefied OTU subset. The Benjamini-Yekutieli method was used to estimate the false discovery rate (FDR) values to control for multiple tests (Asomaning and Archer, 2012). PCoA analyses were performed in R using the ade4 package (Zapala and Schork, 2006). Correlation analysis of the core OTUs was performed using Spearman's rank correlation coefficients, and clustering analysis and heatmap construction were performed in R using the pheatmap package.

## Additional requirements

Nucleotide sequence accession numbers: The sequence data reported in this paper have been deposited in the NCBI database (Accession Numbers: PRJNA412680).

## Author contributions

JZ and YS designed the study. JC and CM collected samples. QP and DH processed and sequenced samples. JZ and SJ analyzed data. QP and SJ wrote the paper.

### Conflict of interest statement

The authors declare that the research was conducted in the absence of any commercial or financial relationships that could be construed as a potential conflict of interest.
