# Supplementary material for: Unique Microbial Diversity and Metabolic Pathway Features of Fermented Vegetables From Hainan, China
Source: Front Microbiol. 2018 Mar 6;9:399. doi: 10.3389/fmicb.2018.00399 (PMC5845746; doi:10.3389/fmicb.2018.00399)
Supplement: Supplementary file 1 [file Presentation1.pdf]

Supplementary Material

Unique features of microbial diversity and metabolic pathways in fermented vegetables in Hainan, China

Qiannan Peng[1#], Shuaiming Jiang[1#], Jieling Chen[1], Chenchen Ma[1], Dongxue Huo[1], Yuyu Shao[2*], Jiachao Zhang[1,3*]

*** Correspondence:**

Corresponding Author:

Jiachao Zhang, College of Food Science and Technology, Hainan University, Haikou 570228, P. R. China

email: zhjch321123@163.com

Yuyu Shao, College of Food Engineering and Nutritional Science, Shaanxi Normal University, Xi'an 710119, Shaanxi, P. R. China

email: icheesemaking@yahoo.com

1 Supplementary Figures and Tables

1.1 Supplementary Table

Region		Type	Region		Type
ES	Wanning	FCC (n=3)	WS	Lingao	FCC (n=1)
		FGB (n=2)			FBS (n=3)
	Wenchang	FCC (n=4)			FW (n=1)
		FGB (n=1)		Changjiang	FBS (n=1)
MS	Qiongzhong	FGB (n=4)			FCC (n=2)
		FW (n=1)			FW (n=2)
	Wuzhishan	FCC (n=1)		Baisha	FBS (n=4)
		FGB (n=4)			FW (n=1)
		FW (n=1)			

Supplementary Table S1 The regions and types of fermented vegetable samples

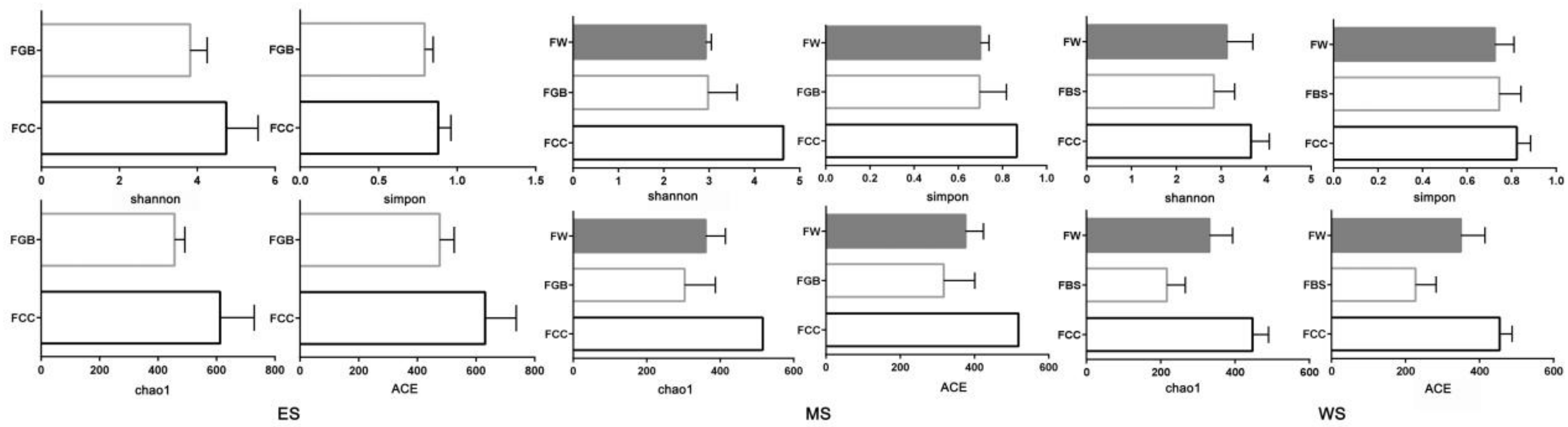

Supplementary Figure S1 Differences in microbial alpha diversity among different types of fermented vegetables in the same region

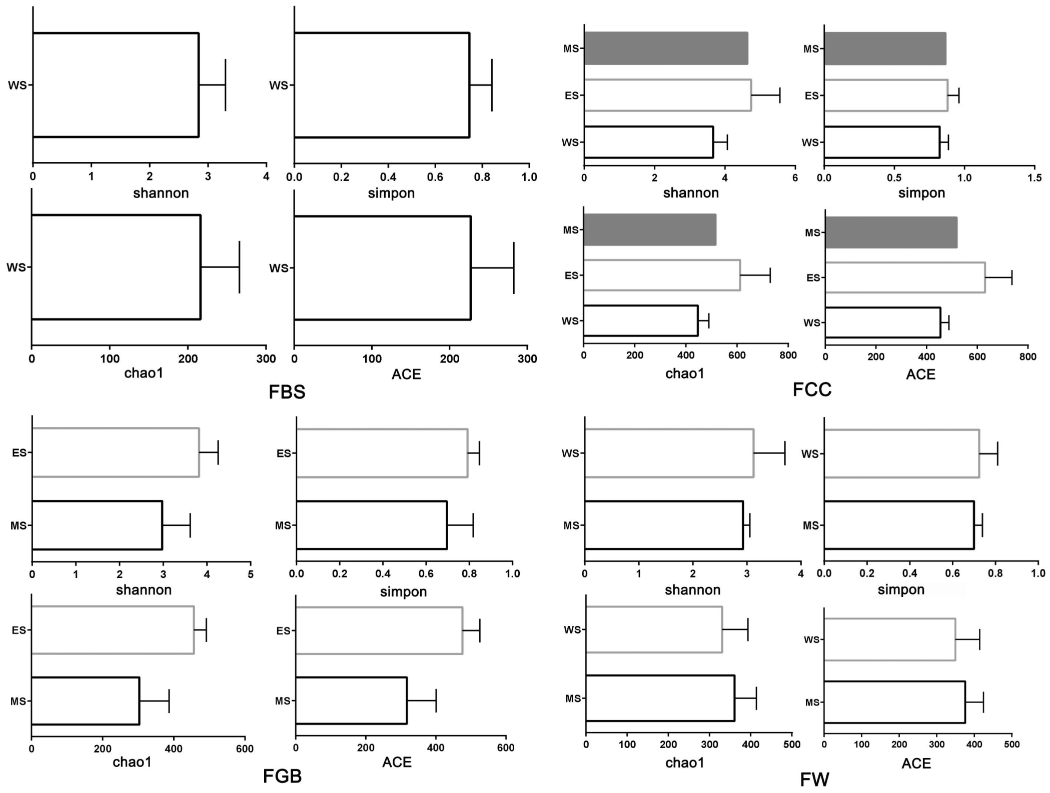

Supplementary Figure S2 Differences in microbial alpha diversity among the same type of fermented vegetables in different regions

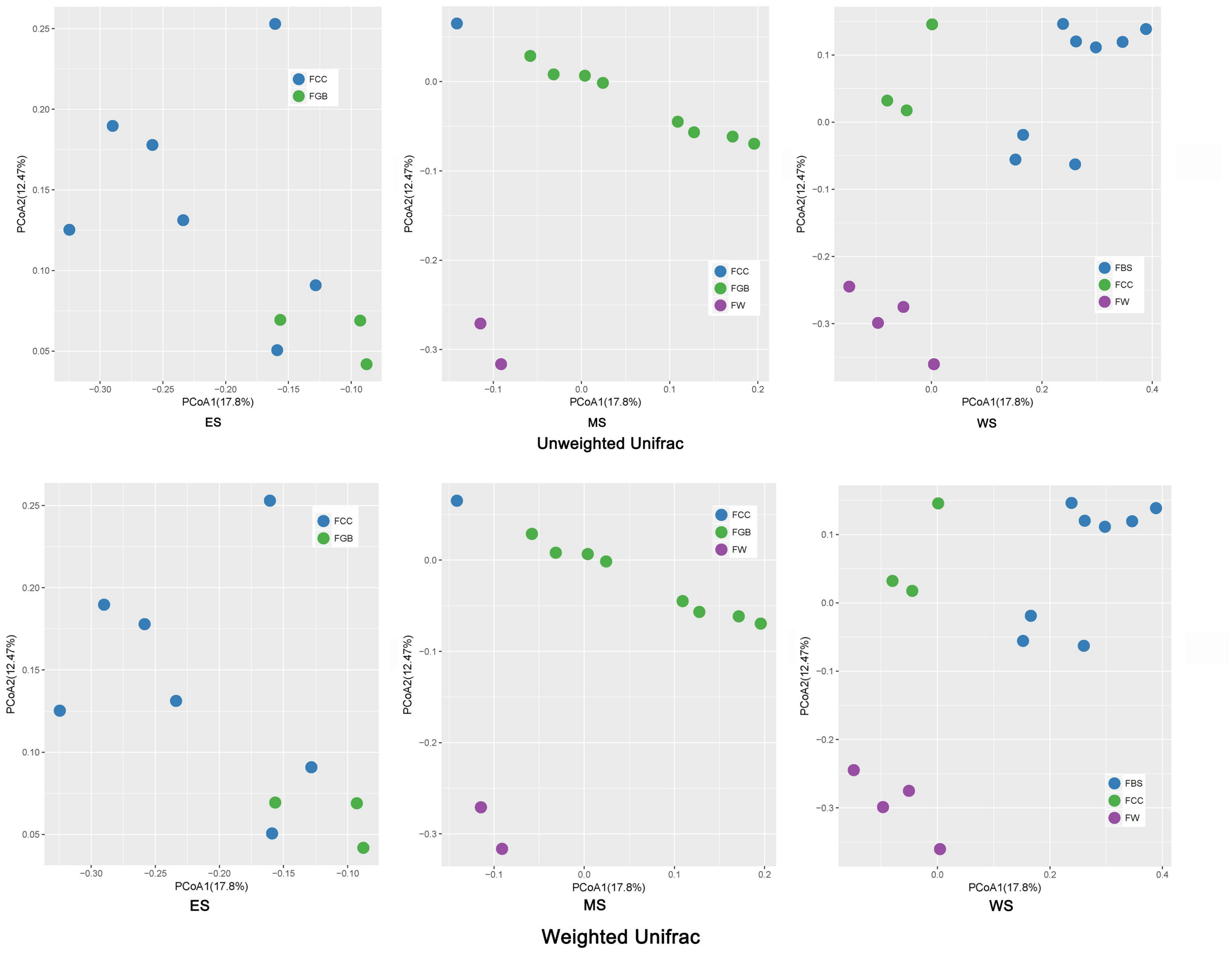

Supplementary Figure S3 Microbial beta diversity among different types of fermented vegetables in the same region

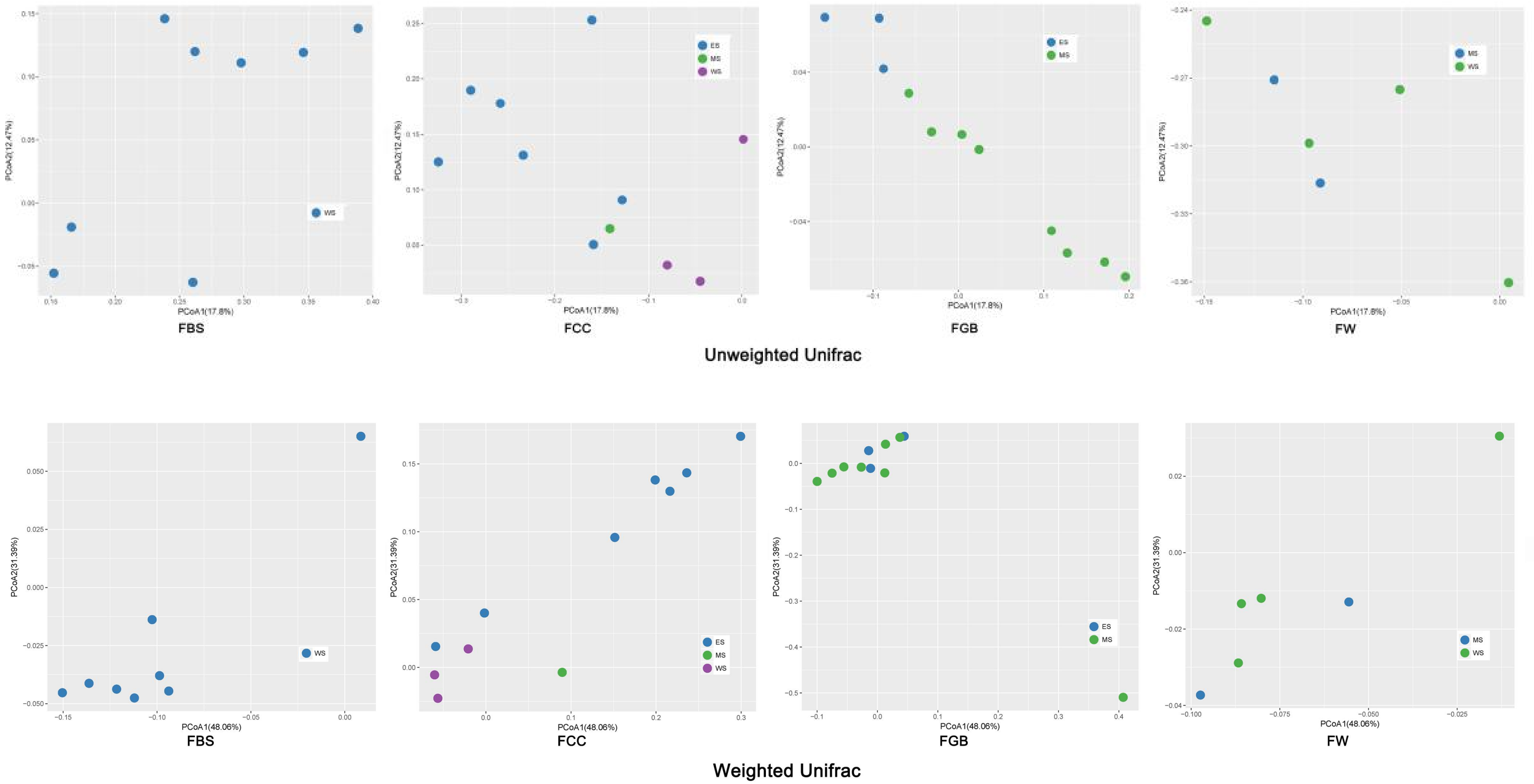

Supplementary Figure S4 Microbial beta diversity among he same type of fermented vegetables in different regions

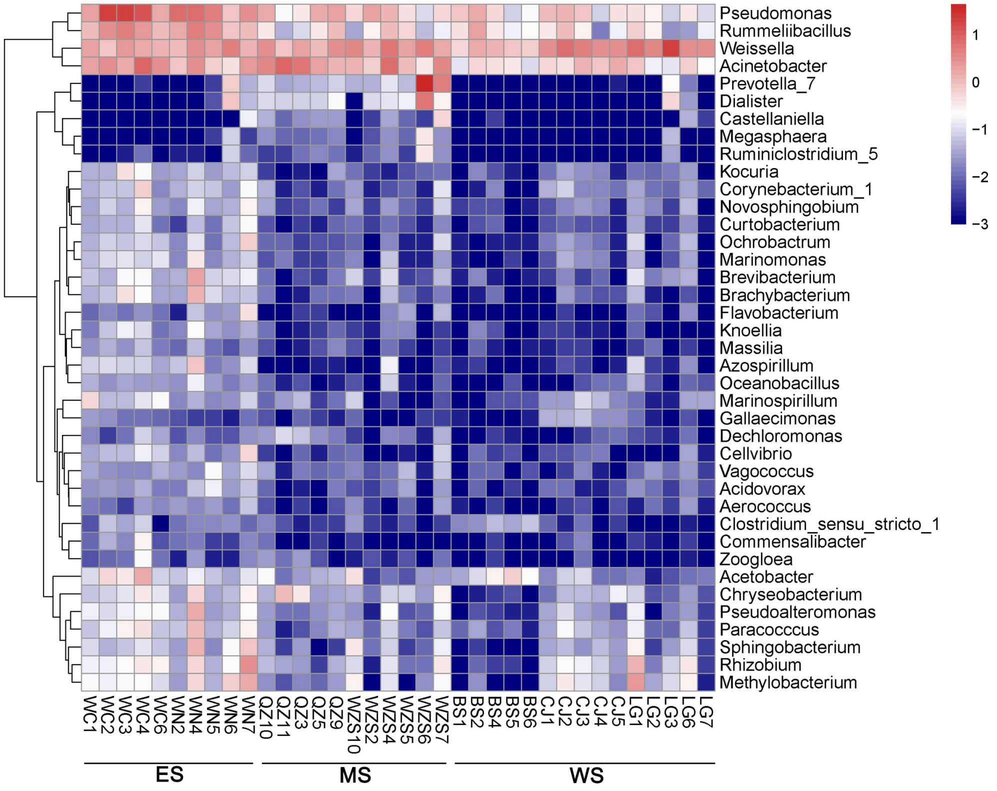

Supplementary Figure S5 Microbial differences at the genus level in different regions

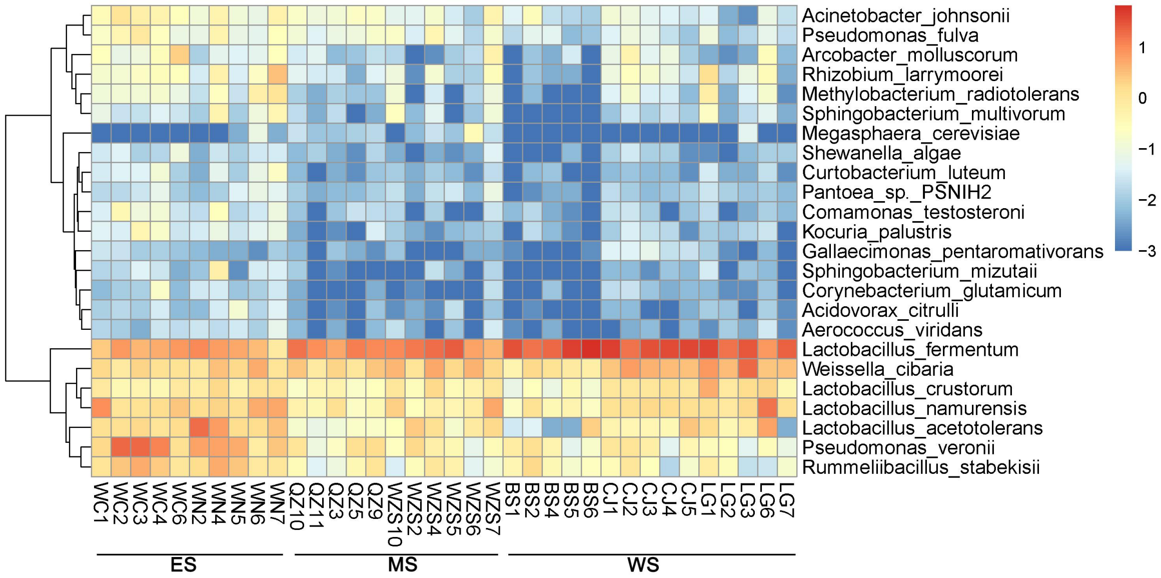

Supplementary Figure S6 Microbial differences at the region level in different regions
